# Establishment of a Mass Concrete Strength-Monitoring Method Using Barium Titanate–Bismuth Ferrite/Polyvinylidene Fluoride Nanocomposite Piezoelectric Sensors with Temperature Stability

**DOI:** 10.3390/s24144653

**Published:** 2024-07-18

**Authors:** Guoliang Lin, Dongwang Lu, Bowen Cui, Aoxiang Lin, Minyi Liu, Yongbin Ye

**Affiliations:** 1 Fujian Provincial Key Laboratory of Advanced Technology and Information in Civil Engineering, Fuzhou 350118, China; gllin@fjut.edu.cn (G.L.); memory946898936@sina.com (D.L.); 2 College of Ecological Environment and Urban Construction, Fujian University of Technology, Fuzhou 350118, China; bowencui120@gmail.com (B.C.); 19841522@fjut.edu.cn (M.L.); 3 College of Civil Engineering, Fuzhou University, Fuzhou 350118, China; 4 Xingyan Group, Fuzhou 350118, China; 13400669800@139.com

**Keywords:** mass concrete, temperature stability, BT–BFO/PVDF, strength monitoring

## Abstract

Mass concrete is widely used in large-scale projects, including metro upper cover structures, water conservancy dams, and heavy equipment foundations, among others, necessitating the process of health monitoring in mass concrete construction. The development of reliable and simple strength-monitoring methods for mass concrete is challenging because the inner temperature of mass concrete is high and changes a lot. This study proposes a strength-monitoring approach for mass concrete using barium titanate–bismuth ferrite/polyvinylidene fluoride (BT–BFO/PVDF) nanocomposite piezoelectric sensors, wherein the new sensors are embedded as actuators and sensors in mass concrete. The stress wave generated by the BT–BFO/PVDF piezoelectric sensors is used to monitor the specimen’s strength for 28 days. The piezoelectric voltage received by the sensors in mass concrete is analyzed. The experimental results indicate that the signal received by the BT–BFO/PVDF sensors is not easily affected by the internal temperature of mass concrete compared with that of the traditional PVDF piezoelectric sensors. The signal parameters sensitive to concrete strength variation and the change trend of concrete strength are closely related to the piezoelectric voltage. Therefore, the proposed approach using BT–BFO/PVDF nanocomposite piezoelectric sensors is efficient (error < 10%) in mass concrete monitoring. Moreover, the monitoring results do not need temperature compensation. The physical meaning of the obtained strength prediction formula is proposed. An experimental system based on PVDF dynamic strain-sensing characteristics is established.

## 1. Introduction

Mass concrete has broad application prospects and a high market demand. It is widely applied in large-scale projects, such as high-rise building foundation slabs, metro upper cover structures, water conservancy dams, heavy equipment foundations, etc., [1]. However, mass concrete releases a large amount of hydration heat during curing. The heat gathered in concrete is less prone to dissipation due to its low thermal conductivity, which leads to an increased inner temperature of concrete [2]. The inner temperature of mass concrete changes during curing. Therefore, the harmful cracks caused by temperature changes and the shrinkage caused by cement hydration are difficult to avoid during the large-volume concrete construction process.

Temperature field monitoring is one of the most important methods of mass concrete monitoring [3]. However, the immediate cause of crack formation in mass concrete is the actual strength of concrete being lower than the thermal stress [4]. This emphasizes the great significance of developing a concrete construction process strength-monitoring method, excluding the influence of temperature. Concrete strength monitoring at various ages has been investigated for decades now, introducing the maturity method [5,6], the electromechanical impedance method [7,8], acoustic emission [9,10], and the stress wave method [11,12]. The advantages of high sensitivity, real-time monitoring, and not being limited by the building structure shape expand the application of the stress wave method with the aid of piezoelectric sensors [13,14].

Piezoelectric sensors are embedded as actuators and sensors in mass concrete, and the pitch–catch application based on stress waves can realize non-destructive concrete strength monitoring [15,16]. The characteristics of piezoelectric materials, such as the electromechanical coupling performance [17], the elastic [18] and piezoelectric coefficients [19], the dielectric constant, and the loss [20] of the piezoelectric sensor, affect the monitoring sensitivity and accuracy. Temperature changes will cause changes in the dipole motion and conductivity and affect the characteristic properties of piezoelectric sensors. The output signal of piezoelectric sensors will experience a large drift with the temperature increase. Thus, a temperature change will further affect sensitivity and stability under various monitoring conditions [21,22]. The temperature compensation method is usually used to correct the monitoring results [23,24,25]. However, the commonly used hardware compensation method has a high debugging difficulty, low accuracy, and poor universality, thereby reducing the monitoring efficiency. By contrast, the temperature compensation algorithm is very complex and time-consuming. It varies with different sensors, complicating its application in practical engineering projects.

PVDF doped with inorganic piezoelectric materials can improve the temperature stability, with the promise of its application in structural health monitoring due to its excellent piezoelectric properties [26], wide frequency response range [27], and high sensitivity [28] to small charges and amplitude responses. However, even though the relationship between the piezoelectric signals and the concrete strength characteristics has been explored using a logarithmic function [29,30,31], the physical meaning of the parameters in the prediction formula is unclear. Therefore, an experimental system based on the PVDF dynamic strain-sensing characteristics has not been established.

Our preliminary experimental results confirmed the good temperature stability of the barium titanate–bismuth ferrite/polyvinylidene fluoride (BT–BFO/PVDF) nanocomposite piezoelectric sensors [32]. In this work, a mass concrete strength-monitoring approach using BT–BFO/PVDF nanocomposite piezoelectric sensors is further proposed, and experiments are conducted to validate its feasibility and accuracy. A pitch and catch pattern using BT–BFO/PVDF sensors is used to monitor the stress wave at the specimen’s target age within 28 days. Monitoring tests are conducted by comparing the piezoelectric voltages monitored using different sensor types at different mass concrete ages under similar temperature conditions. The temperature stability and sensitivity of the BT–BFO/PVDF piezoelectric sensors are compared with those of pure PVDF using different sensor deployments and layouts in mass concrete. In addition, the relationship between concrete strength and the piezoelectric signals based on the experimental results is determined. The physical meaning of the prediction formula is used for a further investigation. Lastly, an experimental system is established based on the PVDF dynamic strain-sensing characteristics.

## 2. Principle of the Strength-Detection Approach for Concrete with the BT–BFO/PVDF Nanocomposite Piezoelectric Sensors

**Figure 1:**
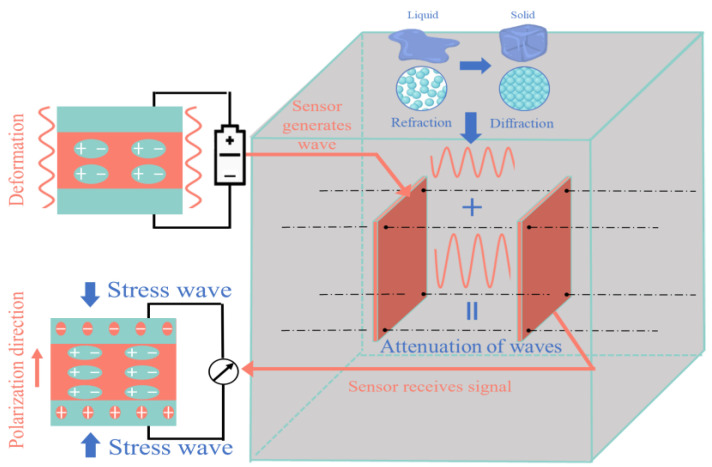
The principle of piezoelectric sensor monitoring in the concrete hydration process.

As shown in Figure 1, the piezoelectric effect causes BT–BFO/PVDF nanocomposite piezoelectric material to produce stress or strain when subjected to an electric field. It also leads to the production of charges when subjected to stress or strain [18]. When the actuator is excited to generate strain, it drives the surrounding particles away from the original position. The surrounding particles then further cause an internal chain reaction that expands in a wave form, consequently forming a stress wave [11]. According to the principle of wave energy attenuation [33], the wave characteristics change as the material properties change during stress wave propagation in the medium. Therefore, the strength development of concrete can be assessed by comparing and analyzing the differences between the signals received by the sensors.

## 3. Specimen Preparation and Test Scheme

### 3.1. Preparation of BT-BFO/PVDF Nanocomposite Piezoelectric Sensors

**Figure 2:**
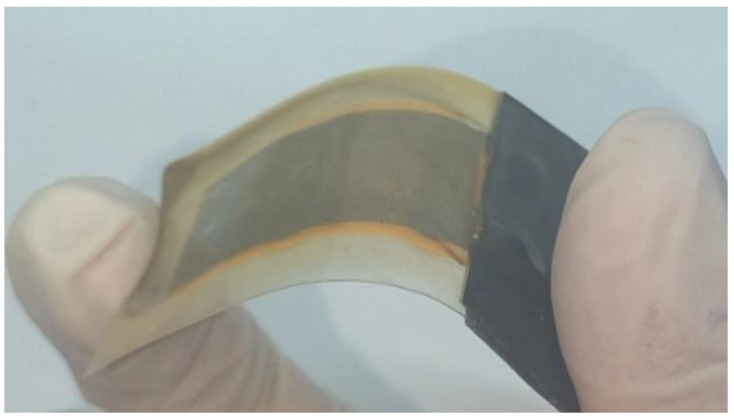
The flexibility test of the sensor after epoxy packaging.

Sandwich-like packaged BT–BFO/PVDF nanocomposite piezoelectric sensors were prepared using a combination of conductive silver cloth and subsequent packaging via a thermoplastic procedure [32]. Figure 2 showed that the previously prepared BT-BFO/PVDF nanocomposite piezoelectric sensor was coated with a layer of epoxy resin via a dip-coating technique, which played a waterproof role, while ensuring the sensor still had good flexibility.

### 3.2. Concrete Specimen

To verify the feasibility of BT-BFO/PVDF nanocomposite piezoelectric sensor monitoring concrete strength, 36 standard square specimens with a side length of 150 mm were prepared for the compressive test and the strength-monitoring test at room temperature. Detailed information of the concrete proportion of the specimen is shown in Table 1, which complied with the Chinese National Standard JGJ 55-2011 [34] (specification for mix proportion design of ordinary concrete).

### 3.3. The Compressive Test and the Strength-Monitoring Test of Concrete Specimens

**Figure 3:**
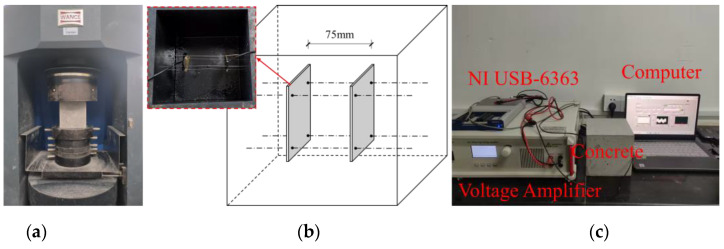
(**a**) Compressive strength test (**b**) position diagram of the piezoelectric sensors inside concrete and (**c**) setup of the pitch-catch system.

In Figure 3a, in order to obtain the compressive strength of concrete, three specimens were randomly selected on the 1st, 2nd, 3rd, 4th, 5th, 6th, 7th, 10th, 14th, 21st, and 28th days, respectively, and a 300-ton oil/electric hybrid servo pressure testing machine was used for the compressive test, which complied with the Chinese National Standard GB/T 50081-2019 [35] (Standard for Test Method of Physical and Mechanical Properties of Concrete). In addition, three specimens were selected to embed sensors inside, as shown in Figure 3b,c, showing the strength-monitoring device at room temperature.

### 3.4. Mass Concrete

To verify the feasibility of concrete strength monitoring using the sensor under high temperatures, an experiment was performed with mass concrete (1000 mm × 1000 mm × 1000 mm). The concrete mix ratio used in the test is shown in Table 1. The specimen-making process is shown in Figure S1. In this experiment, five of the concrete surfaces were supported by wooden boards, and the top surface was covered with heat-insulating and moisturizing materials (polyurethane) for the subsequent heat- and moisture-insulating treatment. The specimen was poured in layers by specialized workers, which complied with the Chinese National Standard GB 50496-2018 [36] (Construction standard for mass concrete).

### 3.5. Layout of Thermocouples and Piezoelectric Sensors in Mass Concrete

**Figure 4:**
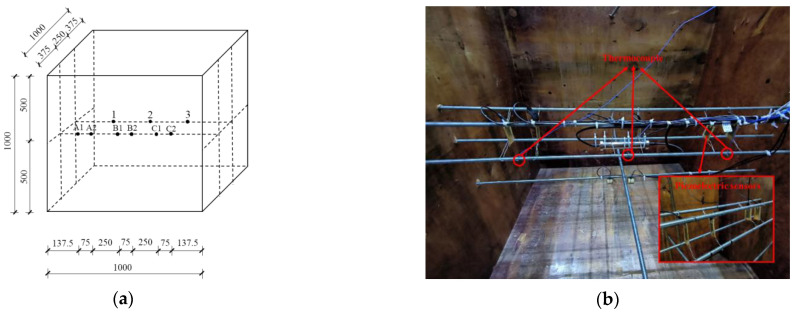
(**a**) Scheme and (**b**) Position diagram of the piezoelectric sensors and the thermocouples inside mass concrete.

The temperature distribution and variation law of mass concrete in the pouring and later curing processes were clarified according to a previous study [32]. The temperature near the center point of mass concrete was the highest. The closer were to the center of the concrete, the higher the concrete temperature was. This indicates the large temperature gradient of mass concrete. A pair of BT–BFO/PVDF nanocomposite piezoelectric sensors (i.e., B1 and B2) were set near the center of mass concrete to analyze the influence of temperature on actual sensor monitoring. A pair of composite piezoelectric sensors (i.e., A1 and A2) were arranged far away from the center point (325 mm) to reduce the interaction of each sensor group. Traditional PVDF sensors (i.e., C1 and C2) were arranged to achieve symmetry with the A1 and A2 sensors, which could be used to analyze the differences between pure PVDF and BT–BFO/PVDF. The pre-embedded sensors were tied with steel wires before pouring. A wire passed through the four corners of the sensor without touching the piezoelectric film and was fixed to a steel bar, preventing sensor displacement during the pouring process and blocking the signal interference and error of the test results. Thermocouples (IKOMAX, Munich, Germany) were embedded near each sensor group to measure the temperature variation in the measuring point (i.e., numbered as 1, 2, and 3). Figure 4 depicts the specific installation position.

### 3.6. A Strength-Detection System for Mass Concrete Supported by Piezoelectric Sensors

**Figure 5:**
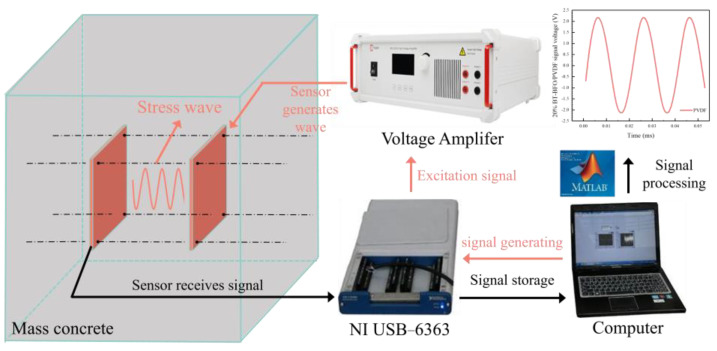
The diagram of mass concrete strength-monitoring device.

A strength-detection system for mass concrete was established using piezoelectric sensors in a pitch and catch pattern, as shown in Figure 5. The voltage excitation signal applied to the actuator was generated by a function waveform generator, and the stress wave propagating from the piezoelectric actuator was received by a sensor at a certain distance. A data acquisition system (NI USB-6363) was employed to record the response in voltage. Monitoring started immediately after concrete pouring. During the monitoring process, the data of each age were recorded, and the corresponding measuring points and ambient temperature were noted. The monitoring intervals were 30 min for the first 7 days and 1 h for the 7th to 28th days to control the experimental error. All test instruments should be powered on for half hours in advanced to reduce the detection error of the instrument. During the detection process, the A1, B1, and C1 sensors were used for excitation, and the A2, B2, and C2 sensors were used for receiving. The sampling frequency set by the NI data acquisition system was 2 MHz. After the actuator was excited for 2 s, the receiver began to collect the signal for 1 s. The sampling interval was 5 s for three tests.

In this study, the input voltage signal was sinusoidal signal with a frequency of 100 kHz, which is expressed by the following Equation (1).

(1) $$V\left(t\right)=V_{0}\sin\left(2\pi ft\right)$$

where $V\left(t\right)$ represents the voltage excitation, $V_{0}$ is the excitation signal amplitude, t refers to the time variable, and f is the frequency adopted.

In order to detect the concrete strength clearly, the selection of a suitable frequency and amplitude of the signal was critical. According to previous research [32], the frequency of the excitation signal was selected as 100 KHz. The amplitude of excitation signal should be high enough to make sure the stress wave can be caught.

## 4. Results and Discussion

### 4.1. Effort of the Excitation Signal Voltage

The pitch–catch mode in piezoelectric signal transmission inevitably caused the loss of some energy during the experiment. To obtain the optimal reception signal, the excitation signal frequency was fixed at 100 kHz, and the excitation voltage signal was gradually increased from 1 to 10 V. In Figure S2, the received signal was relatively small and vulnerable to external environmental noise when the excitation voltage was less than 3 V. The signal received by the composite piezoelectric sensor raised to the maximum (2.5 V), with the excitation voltage increasing from 5 to 9 V. However, the received signal no longer remarkably increased as the excitation voltage increased to 10 V. Therefore, the excitation voltage used in the subsequent experiments on piezoelectric performance was set at 10 V.

### 4.2. Concrete Specimen Analysis

(1) Compressive strength

Compression tests were performed to determine the strength of the concrete specimens at different curing ages. Three specimens were tested for each curing age. The abnormal results were removed. The average of the measured values was reported as the final strength (Table S1). The compressive strength was in accordance with the design requirements.

**Figure 6:**
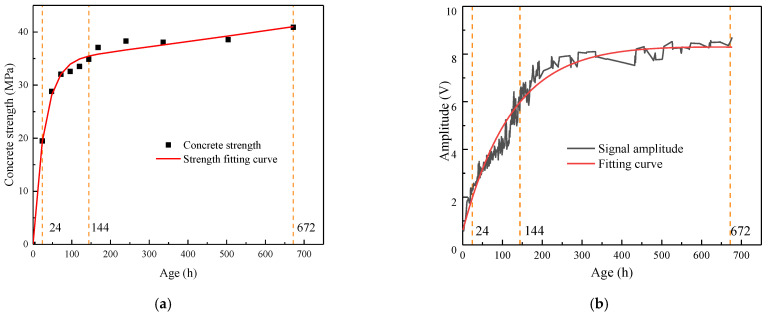
(**a**) Compressive strength development and (**b**) change in the received voltage signal and fitting curve throughout the 28-day curing duration.

Figure 6a represents the compressive strength on the concrete specimens and curve fitting. Assuming that the initial compressive strength of concrete was 0 MPa, the compressive strength rapidly increased on the first day, albeit relatively slowly from the second day to the sixth day. Approximately 85% of the strength development was completed within 6 days. The compressive strength almost did not grow from the 14th day to the 28th day. This phenomenon conformed to the concrete strength development laws, indicating that the compressive strength results can be used for subsequent analysis.

(2) Piezoelectric Signal of the BT–BFO/PVDF Piezoelectric Sensor Used in the Concrete Specimen

The piezoelectric signal detected at each age (0–672 h) within 28 days under standard curing was measured for each concrete specimen. The abnormal signals were eliminated. The average of the three sets of experimental results was calculated as the final piezoelectric signal, and then fitted using the least squares method of the exponential function (Figure 6b).

Figure 6b displays three stages of the increasing trend of the received piezoelectric signal, similar to those of compressive strength with age. In the first stage, the piezoelectric signal rapidly increased from 0.645 to 2.262 V on the first day. Significantly increased stiffness was accompanied by the phase state of concrete, which changed from a liquid to a solid, leading to a stronger guided wave ability of concrete and a higher output voltage of the piezoelectric sensor. In the second stage, the piezoelectric signal grew relatively slowly from the second day to the sixth day, similar to strength growth. During this period, the growth of the concrete hydration products reached a certain degree. Concrete strength growth slowed down. Almost all the concrete had been converted to solid by the seventh day. In the third stage, the piezoelectric signal was nearly constant from the seventh to the twenty-eighth day. This experiment entered the concrete hydration deceleration period, and the internal structure was basically stable. The consistency between the piezoelectric signal and the compressive strength indicated that the BT–BFO/PVDF nanocomposite piezoelectric sensor can be used in concrete strength monitoring.

### 4.3. Temperature Stability of the BT–BFO/PVDF Piezoelectric Sensor

**Figure 7:**
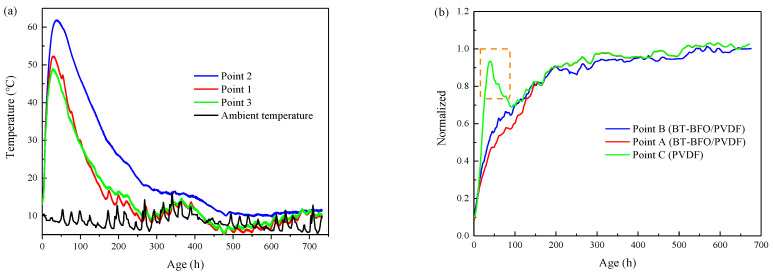
(**a**) Temperature curve of the mass concrete internal measuring point and environment and (**b**) comparison of the signal amplitudes received by each sensor group after normalization.

Mass concrete internal temperature monitoring aims to confirm the temperature variation in the dissipation process of the concrete hydration heat. The ambient temperature was synchronously collected. Figure 7a depicts the mass concrete internal temperature detected by the thermocouple at different positions.

The main monitoring results are as follows: (1) the highest internal temperature in the center of mass concrete was 61.9 °C, which appeared 37 h after pouring; (2) the measuring points at both ends (52.3 °C at Point 1 and 49 °C at Point 3) climbed to the temperature peak before the central measuring point (61.9 °C at Point 2) 28 h after pouring, thereby reaching more than 10 °C of disparity between the central and end points; and (3) the maximum disparity between the ambient (maintained at 10 °C–15 °C) and internal temperatures reached 50 °C, gradually leveling off until 200 h. In other words, the internal temperature of mass concrete was high and sharply changed in the first seven days.

To concentrate the effect of the internal temperature on the piezoelectric signal, the received voltage signal of different sensor types was smoothed and normalized, with the 28-day signal amplitude used as the reference (Figure 7b). The BT–BFO/PVDF piezoelectric sensors at the center and measuring Point 1 showed a similar trend despite the temperature disparity. This was consistent with the variation pattern of the concrete specimen shown in Figure 6b. However, the piezoelectric signal of the traditional PVDF sensor peaked at 38 h of mass concrete ageing, which was close to the time of the maximum temperature of measuring Point 3. The piezoelectric signal coincided with the temperature peaks, indicating that the traditional PVDF was sensitive to the temperature changes. Moreover, strength monitoring was greatly affected by the temperature when PVDF was used as a sensor.

The piezoelectric signals of the concrete specimens at 40 °C–120 °C were detected to further confirm the temperature stability of the BT–BFO/PVDF nanocomposite piezoelectric sensor (Figure S3). The piezoelectric signal fluctuation was less than 7% and did not need modification in the 40 °C–120 °C temperature range. The strength, and not the temperature, was the main influencing factor affecting the monitoring piezoelectric signal. Therefore, the BT–BFO/PVDF nanocomposite piezoelectric sensor processes showed excellent temperature stability.

### 4.4. Monitoring Accuracy of the BT–BFO/PVDF Piezoelectric Sensor

**Figure 8:**
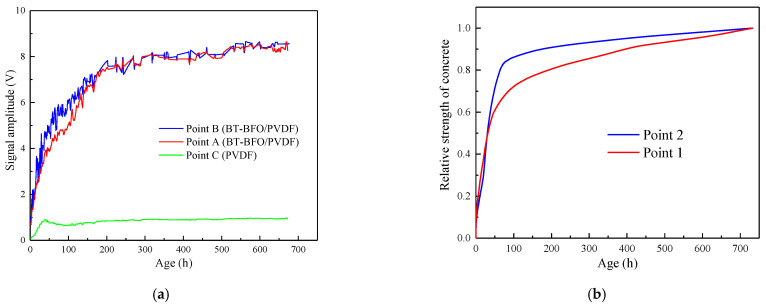
(**a**) Comparison of the signal amplitudes received by each sensor group and (**b**) relative strength curves of Points 1 and 2 ascertained using the maturity method.

The normalized result cannot truly reflect the voltage output caused by the piezoelectric effect. The measured value of the piezoelectric voltage is exhibited in Figure 8a. The piezoelectric voltage output of the traditional PVDF sensor was much smaller than that of the BT–BFO/PVDF sensor. In other words, the traditional PVDF was not only sensitive to the temperature change, but also insensitive to the strength changes during the concrete hydration reaction.

*d*_33_ can be considered a parameter affecting the strain under the same electric field strength. In our previous work, *d*_33_ of the BT–BFO/PVDF nanocomposite piezoelectric sensors (16.194 pC/N) was significantly larger than that of the traditional PVDF sensor (2.552 pC/N) [32]. A higher *d*_33_ means a higher strain caused by voltage excitation, leading to a more significant stress wave. The output piezoelectric voltage generated by the sensors when receiving the stress wave also increased, as shown in Equation (2).

(2) $$S=d_{t}E+s^{E}T$$

where $d_{t}$ is the transpose of *d*_33_; $s^{E}$ is the elastic flexibility matrix under a constant electric field; and S, E, and T are the matrices of the strain, electric field strength, and stress, respectively.

The capacitances of the BT–BFO/PVDF nanocomposite piezoelectric and PVDF sensors were 0.1475 and 0.1385 μF, respectively, at 100 kHz (Figure S4). Despite the large capacitance favoring an excellent current output, the sensor found it difficult to maintain a weak electrostatic charge for a long time under low-frequency concrete confinement stress [37]. Therefore, the piezoelectric signal produced by the sensor did not actually originate from the internal stress on concrete. The pitch–catch system ensured that the changes in the phase state during the hydration process in the internal concrete were captured by the sensors. Furthermore, the higher the *d*_33_ value is, the better the dielectric performance is, and the lower the dielectric loss of the BT–BFO/PVDF nanocomposite piezoelectric sensors is [19,20], which can induce a higher sensitivity to the concrete strength development.

The comparison of piezoelectric signals from the same sensor type at different positions showed slight differences in the voltage signals of the two measuring points within 7 days. The voltage signals were not much different after 7 days (Figure 8a). At the same age, within 7 days, the piezoelectric signals at the center (measuring Point B) were slightly higher than those at the end (measuring Point A), leading to the upward protrusion of the piezoelectric signal curve.

Although mass concrete is poured in an integrated manner, the degree of hydration and the strength development in various parts of the interior cannot be completely synchronized. Therefore, the fundamental reason for the slight increase in piezoelectric signals is ascribed to the differences in the hydration reaction processes that macroscopically manifest as a strength difference. However, no method has yet been able to directly measure the internal strength of mass concrete during the hydration process.

Thus, on the premise that the BT–BFO/PVDF sensor is not sensitive to temperature changes, the strength of each internal measuring point at different ages can be obtained by the maturity method [38]. The temperature curves of each point measured by the thermocouples were used to calculate the internal strength and verify the sensor’s monitoring accuracy (Equation (3)).

(3) $$\left\{\begin{array}{c} M_{L}=\sum K_{i}\left(T_{i}+10\right)\times t_{i}\\ f_{cu,1}{=10.76M_{L}}^{0.331} \end{array}\right.$$

where $M_{L}$ is maturity (℃×d); $T_{i}$ and $t_{i}$ are the temperature and time of concrete curing, respectively; $f_{cu,1}$ is the relative concrete strength (≤100%); and $K_{i}$ is the temperature influence coefficient (Table S2).

Figure 8b illustrates the strength development curve of each measuring point in mass concrete using the maturity method. The calculated internal strength of Point B was higher than that of Point A. The early strength development and temperature are closely related. A higher temperature meant a more intense hydration reaction, which promoted the early strength development of concrete. The slight difference in internal strength was captured by the BT–BFO/PVDF sensor, verifying the sensor monitoring accuracy. Furthermore, the piezoelectric signal of different measuring points gradually tended to become consistent after 7 days (Figure 8a), indicating a relatively uniform internal concrete strength.

### 4.5. Strength-Monitoring Model of Mass Concrete Proposed Using BT–BFO/PVDF Sensors

Due to its high strength-monitoring accuracy and strong temperature stability, the BT–BFO/PVDF nanocomposite piezoelectric sensor can be applied in the strength monitoring of mass concrete at different ages without extra temperature compensation.

According to the growth trend and shape of the concrete strength–signal amplitude scatter plot, a logarithmic function was used to fit these data, as shown in Table 2 and Figure S5. The correlation model established between the strength and the piezoelectric signal can be applied to mass concrete monitoring.

$$y=0.3285\ln\left(0.157589x\right)+1.5667$$

where y and x represent the concrete strength and piezoelectric signal ratios, respectively.

The Poisson’s ratio of C40 concrete was 0.33, as calculated using the formula of Poisson’s ratio in Equation (4), which was close to parameter *a* (0.3285). The physical meaning of parameter *a* might be related to the Poisson’s ratio of concrete.

Parameter *b* is attributed to the correction factor of different piezoelectric sensors. The calculation formula for this is presented as Equation (4). Based on previous research [32], the *d*_33_ values of the BT–BFO/PVDF and PVDF sensors were 16.194 and 2.552 pC/N, respectively. Parameter *b* was calculated as 0.1576, a value close to the fitting results.

(4) $$b=\frac{d_{33,PVDF}}{d_{33,A}}$$

where $d_{33,A}$ and $d_{33,PVDF}$ are the piezoelectric coefficients of the BT–BFO/PVDF nanocomposite piezoelectric sensor and the PVDF sensor, respectively.

Parameter *C* may be identified as the corrected coefficient of the concrete strength.

Equation (5) was used to calculate the error δ of the strength prediction formula to verify its accuracy.

(5) $$\delta =\frac{M-P}{P}\times 100\%$$

where M is the measured compressive strength of concrete, and P is the predicted compressive strength value. Figure S6 depicts the error analysis of the compressive strength prediction formula. The maximum error did not exceed 10%.

## 5. Conclusions

In this study, we proposed a concrete strength-detection approach using the actuating and sensing technologies of BT–BFO/PVDF nanocomposite piezoelectric sensors under a high-temperature environment. Two different concrete specimens were constructed and experimentally investigated to demonstrate the feasibility and the accuracy of the proposed method. The influence of hydration heat on the sensor was studied, and sensor’s sensitivity to the mass concrete strength was verified. In addition, the relationship between the signal amplitude received by the BT–BFO/PVDF nanocomposite piezoelectric sensor and concrete strength was established. The following conclusions are drawn based on this experimental study:

(1) The pitch–catch system was used to extract the signal parameters that were sensitive to the concrete strength variation. The results showed that the change trend of concrete strength was closely related to that of the signal amplitude. The main reason for this was that the compressive strength of concrete is associated with the hydration reaction. The hydration product-transformed concrete transferred from a liquid to a solid, promoting stress wave transmission. Therefore, the signal amplitude change in the BT–BFO/PVDF nanocomposite piezoelectric sensors can be used to detect the compressive strength of concrete with age variation.

(2) Compared with that received by the traditional PVDF piezoelectric sensor, the signal amplitude curve received by the BT–BFO/PVDF nanocomposite piezoelectric sensor did not show an obvious peak point under the hydration heat of mass concrete. In other words, the temperature stability improvement made the BT–BFO/PVDF nanocomposite piezoelectric sensor immune to hydration heat. The higher the *d*_33_ value is, the better the dielectric performance is, and the lower the dielectric loss of the BT–BFO/PVDF nanocomposite piezoelectric sensor is, which can capture slight differences in mass concrete strength.

(3) A concrete strength prediction formula was proposed using BT–BFO/PVDF nanocomposite piezoelectric sensor monitoring to evaluate the mass concrete strength at different ages. The results showed a good correlation between the concrete strength and the sensor signal. The fitting correlation coefficient (R) reached 0.9801. Analyzing the physical meaning of each coefficient in the prediction formula led us to determine that the Poisson’s ratio of concrete and the *d*_33_ value of new sensors were the main factors affecting concrete strength monitoring. The error between the calculated and measured concrete strength values was within 10%, indicating that the concrete strength prediction formula can be used to effectively evaluate the mass concrete strength at different ages.

In summary, the proposed approach of embedding BT–BFO/PVDF nanocomposite piezoelectric sensors inside mass concrete can detect concrete strength under high temperatures. When the prediction formula for concrete strength was established, only the physical meaning of the parameters in the formula was preliminarily judged. More detailed research on this aspect can be performed in the future. Further investigation on the application of sensors in mass concrete after 28 days of curing can also be conducted to prevent the idleness of the embedded sensor.

## Figures and Tables

**Table 1:** Detailed concrete proportion (kg/m^3^).

Strength Grade	Cement	Water	Fine Aggregate	Coarse Aggregate
C40	436	220	542	1152

**Table 2:** Fitting curve parameters and correlation coefficient.

Fitting Function	Correlation Coefficient (R)	*a*	*b*	*c*
*y = a* × *ln*(*b* × *x*) *+ c*	0.9801	0.3285	0.157589	1.5667

## Data Availability

Data will be made available on request.

## Supplementary Materials

The following supporting information can be downloaded at: https://www.mdpi.com/article/10.3390/s24144653/s1. Table S1. Compressive strength at different ages, Table S2. Values of the temperature influence coefficient, Figure S1. Mass concrete specimen, Figure S2. Received voltage signals of the BT–BFO/PVDF nanocomposite piezoelectric sensor under different excitation voltages, Figure S3. Signal amplitude received by the BT–BFO/PVDF nanocomposite piezoelectric sensor under different temperatures, Figure S4. Comparison of the capacitances of different sensor types, Figure S5. Concrete strength–signal amplitude scatter plot and fitting curve, Figure S6. Error analysis of the compressive strength prediction formula.
