# Supplementary material for: Establishment of a Mass Concrete Strength-Monitoring Method Using Barium Titanate–Bismuth Ferrite/Polyvinylidene Fluoride Nanocomposite Piezoelectric Sensors with Temperature Stability
Source: Sensors (Basel). 2024 Jul 18;24(14):4653. doi: 10.3390/s24144653 (PMC11280568; doi:10.3390/s24144653)
Supplement: Supplementary file 1 [file sensors-24-04653-s001.zip › sensors-3079400-supplementary.pdf]

Table S1. Compressive strength at different ages

**Age (d)**	**Age (h)**	**Average values (MPa)**	**Strength percentage (/%)**
0	0	0	0.00
1	24	19.48	47.63
2	48	28.81	70.46
3	72	32.03	78.33
4	96	32.58	79.69
5	120	33.50	81.92
6	144	34.87	85.27
7	168	37.08	90.69
10	240	38.30	93.66
14	336	38.06	93.08
21	504	38.57	94.34
28	672	40.89	100.00

Table S2. Values of the temperature influence coefficient

Temperature range (/℃)	K_i	temperature range (/℃)	K_i
4~7	0.81	48~52	2.00
8~27	1.00	53~54	3.60
28~32	1.15	55~56	5.30
33~42	1.29	57~59	7.60
43~47	1.34	60~67	8.50

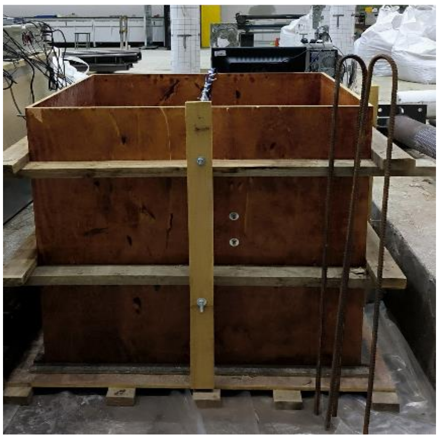

Figure S1. Mass concrete specimen

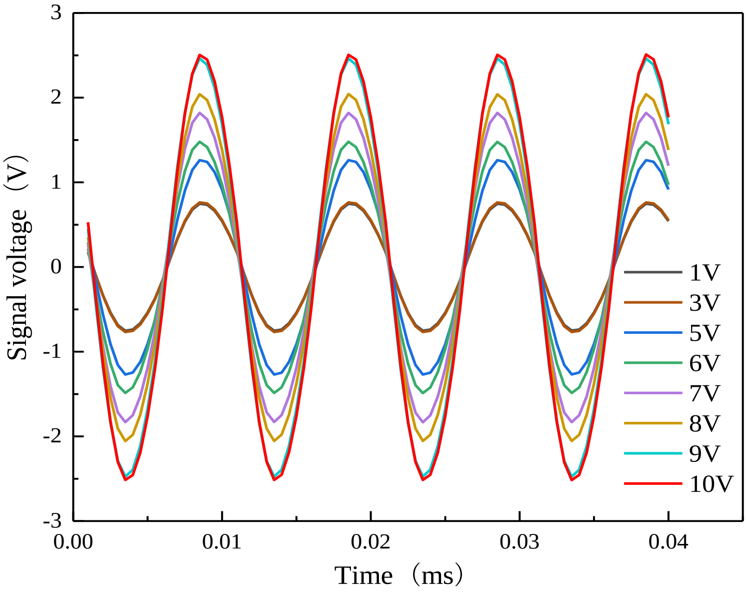

Figure S2. Received voltage signals of the BT–BFO/PVDF nanocomposite piezoelectric sensor under different excitation voltages

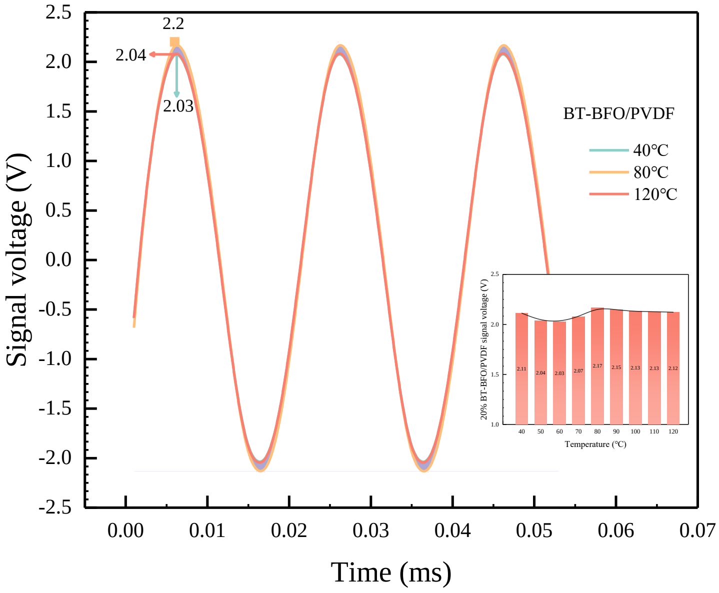

Figure S3. Signal amplitude received by the BT–BFO/PVDF nanocomposite piezoelectric sensor under different temperatures

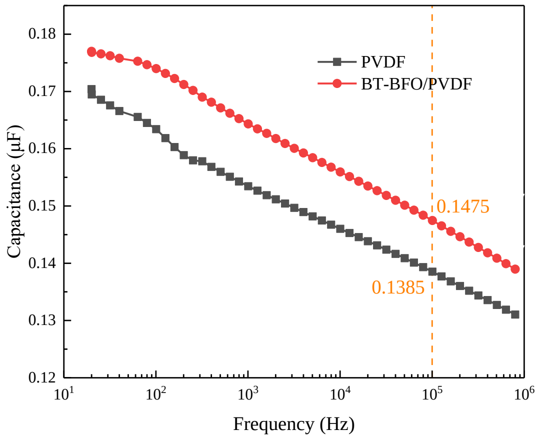

Figure S4. Comparison of the capacitances of different sensor types

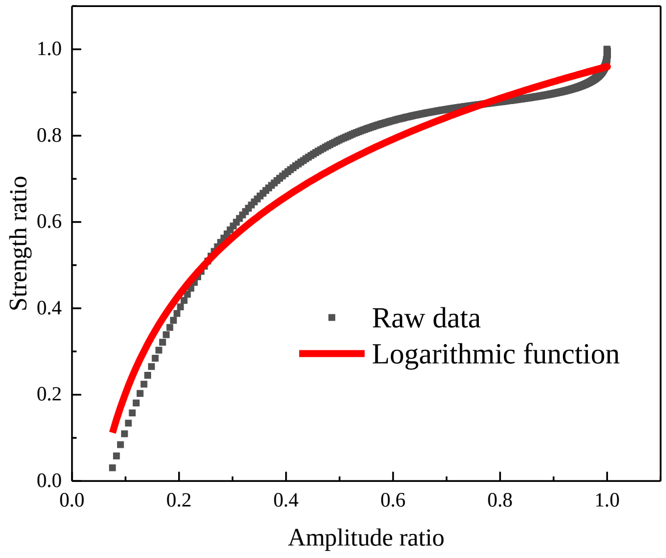

Figure S5. Concrete strength–signal amplitude scatter plot and fitting curve

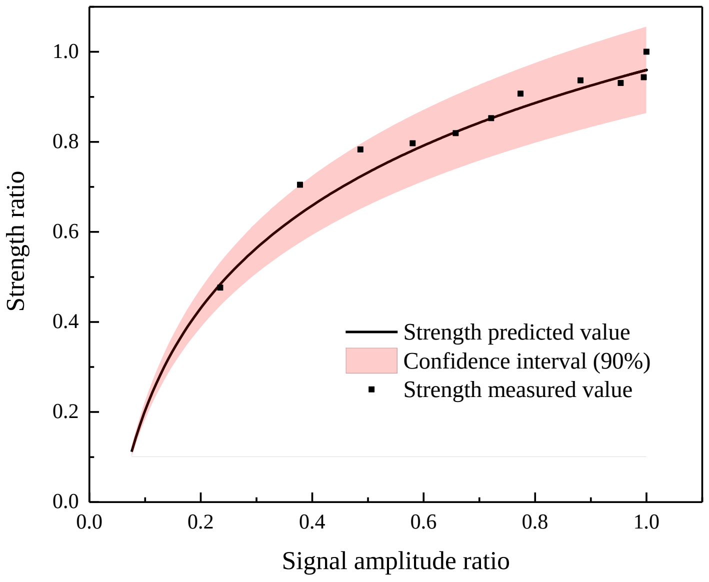

Figure S6. Error analysis of the compressive strength prediction formula
